# Scientific developments in understanding food allergy prevention, diagnosis, and treatment

**DOI:** 10.3389/fimmu.2025.1572283

**Published:** 2025-04-22

**Authors:** Shirin Karimi Hund, Vanitha Sampath, Xiaoying Zhou, Bryan Thai, Khushi Desai, Kari C. Nadeau

**Affiliations:** ^1^ Clinic for Internal Medicine, Spital Zollikerberg, Zollikerberg, Switzerland; ^2^ Department of Environmental Health, Harvard T. H. Chan School of Public Health, Boston, MA, United States; ^3^ Geffen Academy at UCLA, Los Angeles, CA, United States

**Keywords:** food allergy, IgE antibodies, prevention, diagnosis, biomarkers, immunotherapy, biologics

## Abstract

Food allergies (FAs) are adverse immune reactions to normally innocuous foods. Their prevalence has been increasing in recent decades. They can be IgE-mediated, non-IgE mediated, or mixed. Of these, the mechanisms underlying IgE-mediated FA are the best understood and this has assisted in the development of therapeutics. Currently there are two approved drugs for the treatment of FA, Palforzia and Omalizumab. Palfornia is a characterized peanut product used as immunotherapy for peanut allergy. Immunotherapy, involves exposure of the patient to small but increasing doses of the allergen and slowly builds immune tolerance to the allergen and increases a patient’s allergic threshold. Omalizumab, a biologic, is an anti-IgE antibody which binds to IgE and prevents release of proinflammatory allergenic mediators on exposure to allergen. Other biologics, investigational vaccines, nanoparticles, Janus Kinase and Bruton’s tyrosine kinase inhibitors, or DARPins are also being evaluated as potential therapeutics. Oral food challenges (OFC) are the gold standard for the diagnosis for FA. However, they are time-consuming and involve risk of anaphylaxis; therefore, alternative diagnostic methods are being evaluated. This review will discuss how the immune system mediates an allergic response to specific foods, as well as FA risk factors, diagnosis, prevention, and treatments for FA.

## Introduction

The prevalence of FA (FA), which are adverse immune reactions to normally innocuous foods, have been increasing in recent decades (1). Adverse reactions can be mild to moderate to severe, and, in rare cases, even fatal. The increasing prevalence of FA and the risk of severe reaction is a cause for concern, affecting quality of life for patients and their families as well as increasing public health burden.

Allergic diseases, such as FA, are hypersensitivity reaction. The traditional Gel and Cooms classification of hypersensitivity reactions categorizes them into types I-IV. FA is a Type I IgE-mediated hypersensitivity disease with a rapid onset from exposure to reaction Type II and Type III hypersensitivity reactions are mediated via IgG/IgM, with Type II reactions associated with complement system activation and type III reactions are associated with immune complex reactions. Type IV hypersensitivity involves a delayed T-cell-mediated reactions (e.g., allergic contact dermatitis) (2, 3). However, with increasing knowledge of the molecular mechanisms underlying hypersensitivity reactions, the classification has now been expanded to include seven main types. This is detailed in the 2023 EAACI position paper (4). While there is still significant work to be done to correlate these different allergy endotypes to clinical phenotypes, these classification are steps towards personalized treatments. It must be recognized that the etiology of many hypersensitivity reactions are mixed and that the mechanism underlying hypersensitivity diseases can often better be explained by a combination of different endotypes.

Individuals with FAs can be allergic to a wide range of foods, however, the most common food allergens include peanuts, tree nuts, soy, fish, shellfish, sesame, milk, and eggs. Symptoms of FA include but are not limited to acute urticaria and angioedema, chest tightness, nausea, vomiting, abdominal pain, and anaphylaxis (5). Interestingly, many patients with FAs often have comorbid atopic diseases and these follow an age-dependent pattern. The first atopic disease to occur is generally AD in early infancy followed by FA, asthma and/or AR at a later stage; this progress of atopic diseases, starting with AD, has been termed the atopic or allergic march (6). Studies have shown that not all children with AD subsequently develop other atopic conditions, however those with moderate to severe AD are at higher risk of developing other atopic diseases (7).

**Figure 1:**
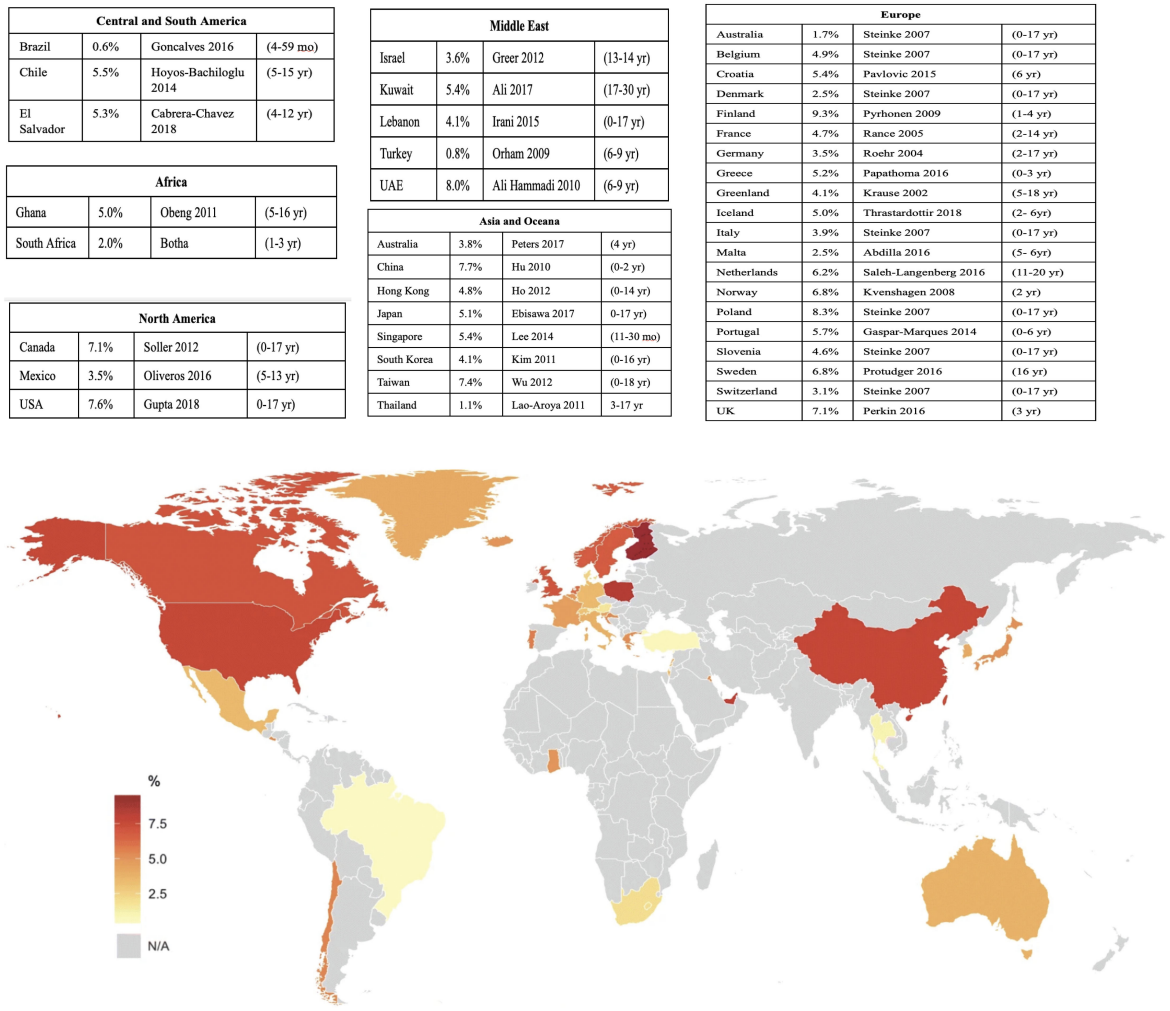
Population-based estimates of current global pediatric FA prevalence. Source: Warren, C. et al. (2020). *Population-based estimates of current pediatric FA prevalence in countries around the world*. Current Allergy and Asthma Reports. https://doi.org/10.1007/s11882-020-0898-7.

Estimates of FA prevalence vary due to differences in methodology, demographics, geographical location, and type of food allergen (1, 8). **Figure 1** shows the wide variations in estimated FA prevalence around the globe. Oral food challenges (OFC) are the gold standard for the diagnosis for FA, however, they are time-consuming and involve risk of anaphylaxis; consequently, there are few challenge-proven data on FAs. Many of the estimations provided in prevalence studies for common allergens have used surveys, measures of IgE (skin prick testing (SPT) or blood tests) and clinical history (9). Self-/parent-proxy surveys have been shown to often overestimate the true prevalence as they likely include food intolerances rather than true FA. Food intolerances include enzyme and metabolic deficiencies, functional defects, pharmacologic effects, psychosomatic effects, sensitivity to food additives or reactions to naturally occurring chemicals or toxins in foods (10). However, surrogate markers of FA such as health service utilization and clinical history, together with allergen-specific immunoglobulin E (sIgE) have provided convincing data that the prevalence of FA is increasing in both Western and developing countries (1). In the US, FA has been estimated to affect between 6.2-10.8% of adults (11). In 2018, another prevalence survey conducted in the US examined over 50,000 households and reported that IgE-mediated FA s affects approximately 11% of children (12). In Australia, data from the population-based, longitudinal HealthNuts study of 6 and 10-year old children indicated FA in 6.4% of 6-year olds and 6.3% of 10-year-olds. Among infants with challenge-confirmed FA in infancy, 45% had persistent disease at age 10 years (13). A 2023 population based study found that among children and adults with FA, an estimated 40% and 48% had multi-FA, respectively (14). Another study from the UK estimated that around 6% of the UK adult population had a clinically confirmed IgE-mediated FA with a spectrum of severity of reaction from mild (like oral itching) to anaphylaxis (15). While allergy to some foods like milk, egg, wheat, and soy are commonly outgrown, allergy to other foods such as peanut, tree nuts, and seafood is typically lifelong (16). 

FAs have become an increasingly worrying concern for clinicians, families, and policymakers in many parts of the world due to their increasing prevalence and risk of severe reaction. While genetics plays a role in the etiology of allergic diseases, the rate of increase in allergic diseases is too rapid to be explained by genetics alone. Changes in lifestyle factors with modernization and increased exposure to environmental pollutants are implicated in these increases. Lifestyle changes in recent decades include increased urbanization, hygiene, use of antibiotics, food emulsifiers, and soaps, consumption of processed foods and decreased outdoor activity, and exposure to pets, farm animals, and green space (17).

This review highlights our current knowledge of the mechanism of IgE-mediated FAs, the differences in immune response between allergic and healthy individuals, and the current knowledge on preventing, diagnosing, and treating FA.

## Cellular and molecular mechanisms: allergic reaction and tolerance in FA

As mentioned above, FAs are classified as Type I hypersensitivity reactions. However, in practice, FAs are generally classified into either IgE-mediated or non-IgE-mediated (cell mediated) (9). While this classification is an oversimplification, it is used for diagnostic and therapeutic purposes. IgE-mediated allergic diseases (also called atopic diseases) are best understood. In addition to FA, IgE-mediated diseases include allergic rhinitis (AR), atopic dermatitis (AD), and asthma. Non-IgE-mediated reactions are typically delayed in onset and occur between 4 and 28 hours after ingestion (1). Unlike IgE mediated FAs which affects multi-organs, the non-IgE mediated FAs primarily affect the gastrointestinal tract (18). Mixed-IgE reactions are conditions associated with FAs involving mechanisms from both IgE and non-IgE-mediated allergies (19). Here, we detail current understanding of mechanisms underlying IgE-mediated FA and immune tolerance to common allergens

Great strides have been made in understanding the molecular pathways underlying FA reactions as well as that of tolerance to innocuous foods. The cellular and molecular mechanisms have been detailed in a number of good reviews and is summarized here (20, 21). This mechanistic understanding has been of great value in the design and development of treatments for FA and other atopic diseases, as they share some common pathways. Some of the drugs currently in use for atopic diseases and the mechanism through which they act are detailed in the section on treatments. It is hypothesized that allergic sensitization is initiated through allergen exposure via a defective skin epithelial barrier and tolerance mediated on oral exposure of food allergen (dual allergen exposure hypothesis) (20, 22).

Loss of epithelial barrier integrity can be caused by allergens, infectious agents (e.g., bacteria, fungi, and viruses), injury, environmental substances (e.g., particulate matter, microplastics, detergents), or genetic predisposition (e.g., filaggrin (FLG), claudin-1). Surfactants, enzymes, and emulsifiers present in processed foods have been shown to damage the epithelial barrier (23, 24). The key role of a disrupted epithelial layer in allergy is supported by the observations that infants with eczema or those with FLG loss-of-function mutation are at much greater risk of FA later in life (25, 26). Other epidermal defects that result in disruption of tight junction on the skin such as claudin-1 gene polymorphisms are also associated with increased risk of FA (27, 28).

**Figure 2:**
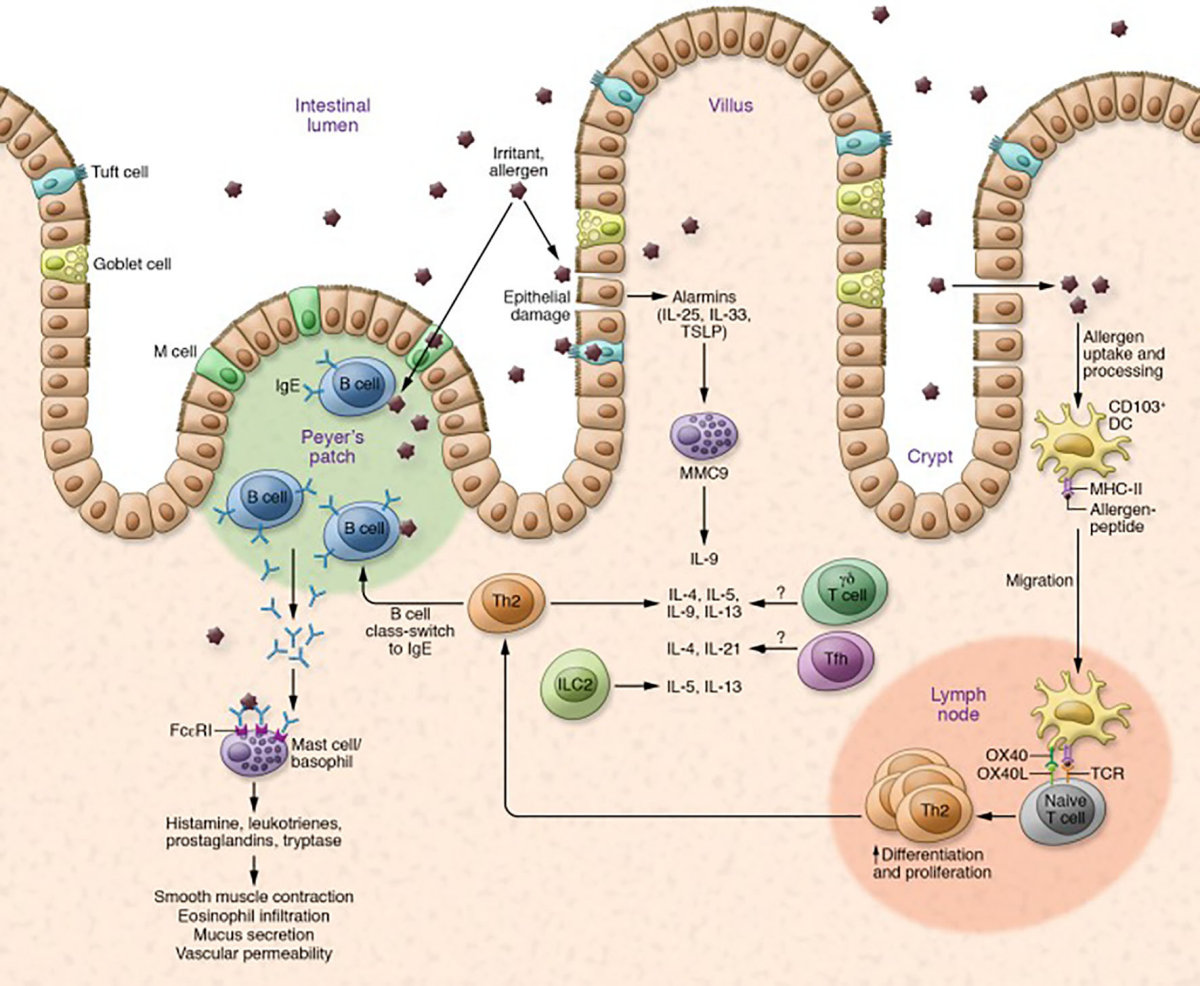
Mechanism of allergic reaction in FA. Allergic reaction is mediated by upregulation of Th2 cytokines and B cell class switching, which leads to production of IgE antibodies and degranulation of mast cells and basophils. Degranulation initiates allergic responses, such as smooth muscle contraction, eosinophil infiltration, mucus secretion, and vascular permeability. Other cells such as Tfh cells, γδ T cells, and ILC2s are also thought to play a role in allergic reactions. Key cytokines involved are IL-25, IL-33, IL-4, IL-5, IL-13, and IL-9. Adapted from: Sampath V, Nadeau KC. Newly identified T cell subsets in mechanistic studies of food immunotherapy. *J Clin Invest*. 2019;129(4):1431-1440. doi:10.1172/JCI124605.

In FA, the TH2 inflammatory cascade leading to allergic-specific IgE production is well characterized. This cascade is initiated by release of proinflammatory cytokines IL-25, IL-33, and Thymic stromal lymphopoietin (TSLP) by disrupted epithelial cells. These epithelial cytokines are collectively called alarmins. These alarmins initiate a pro-inflammatory cascade primarily via dendritic cells and naïve T cells but also via ILC2s (29). IL-33 upregulates OX40L on dendritic cells driving differentiation of naïve CD4+ T cells to inflammatory allergen-specific Th2 cells, which produce proinflammatory cytokines IL-4, IL-5, IL-9, and IL-13 (30). A few studies have also reported increases in TH9 cells which secrete IL-9 in patients with peanut allergy (31). ILC2s also produce the proinflammatory cytokines IL-5, IL-9, and IL-13 (29). These Th2 cytokines mediate IgE class switching by B cells. Allergen-specific IgE cells then bind FcϵRI. Individuals with IgE bound to mast cells or basophils are now sensitized to the food allergen. In FA, sensitized individuals on subsequent exposure to allergens cause the FcϵRI-bound IgE antibodies to crosslink, degranulate, and release allergic mediators such as histamine, leukotrienes, and prostaglandins into the surrounding tissue (**Figure 2**) leading to the symptoms of allergic reactions (5, 32).

While we have some understanding of the pathways associated with allergic reactions to common allergens, our understanding the mechanisms leading to persistence of FAs is incompletely understood (33). The mechanism by which some individuals eventually develop tolerance while others individuals maintain high IgE titers to specific allergens is not fully understood. Allergen-specific CD4^+^T cells have a pivotal role in causing and maintaining the allergic response to food allergens. The high titers have long been thought to be sustained by long-lived IgE+ plasma cells. However, research indicates that lifelong reactivity to specific allergens is conferred by allergen-specific long-lived memory B cells that replenish the IgE+ plasma cell compartment. Further, B-cell reactivation appears to require allergen re-exposure and IL-4 production by CD4 T cells (34).

The mechanism of immune tolerance to foods is an active process with immune training starting during the critical period of early infancy. In tolerance, ingested antigens from food particles do not provoke an immune response. This can be thought of a “normal” response to food intake whereas FA is the “pathologic” response to food exposure. In tolerance, food allergens are detected by dendritic cells present in the epithelial layer of the gut which take up the food antigen and migrate to the mesenteric lymph nodes. Dendritic cells also induce expression of the CCR9 and α4β7 receptors, which are some of the factors that have been shown to induce T cells migration to the gut (30). They also secrete co-stimulatory molecules such as TGF-β, IL-10, retinoic acid, indoleamine 2,3, dioxygenase, and retinal aldehyde dehydrogenase and drive differentiation of T naïve cells into T regulatory cells (Tregs) (30, 35). Tregs are essential for the suppression of Th2-mediated inflammation and induction of immune tolerance. T regs skew B cell class switching to secrete IgA and IgG4. These antibodies block the inflammatory allergic IgE state. Overall, immune tolerance involves differentiation of T naïve cell to Treg cells, decreased production of IgE by B cells, increased IgG_4_ and IgA production by B cells, induction of IL-10 producing dendritic cells, and suppression of basophil, eosinophil, and mast cell activation (35).

While the pathophysiological mechanisms involving the defective skin epithelial barrier and oral intake of food allergens culminating in the allergic inflammatory response are well known, the role of genetics and environmental factors which predispose individuals to the development of FA is still an area of active research.

## Factors associated with FA risk

**Figure 3:**
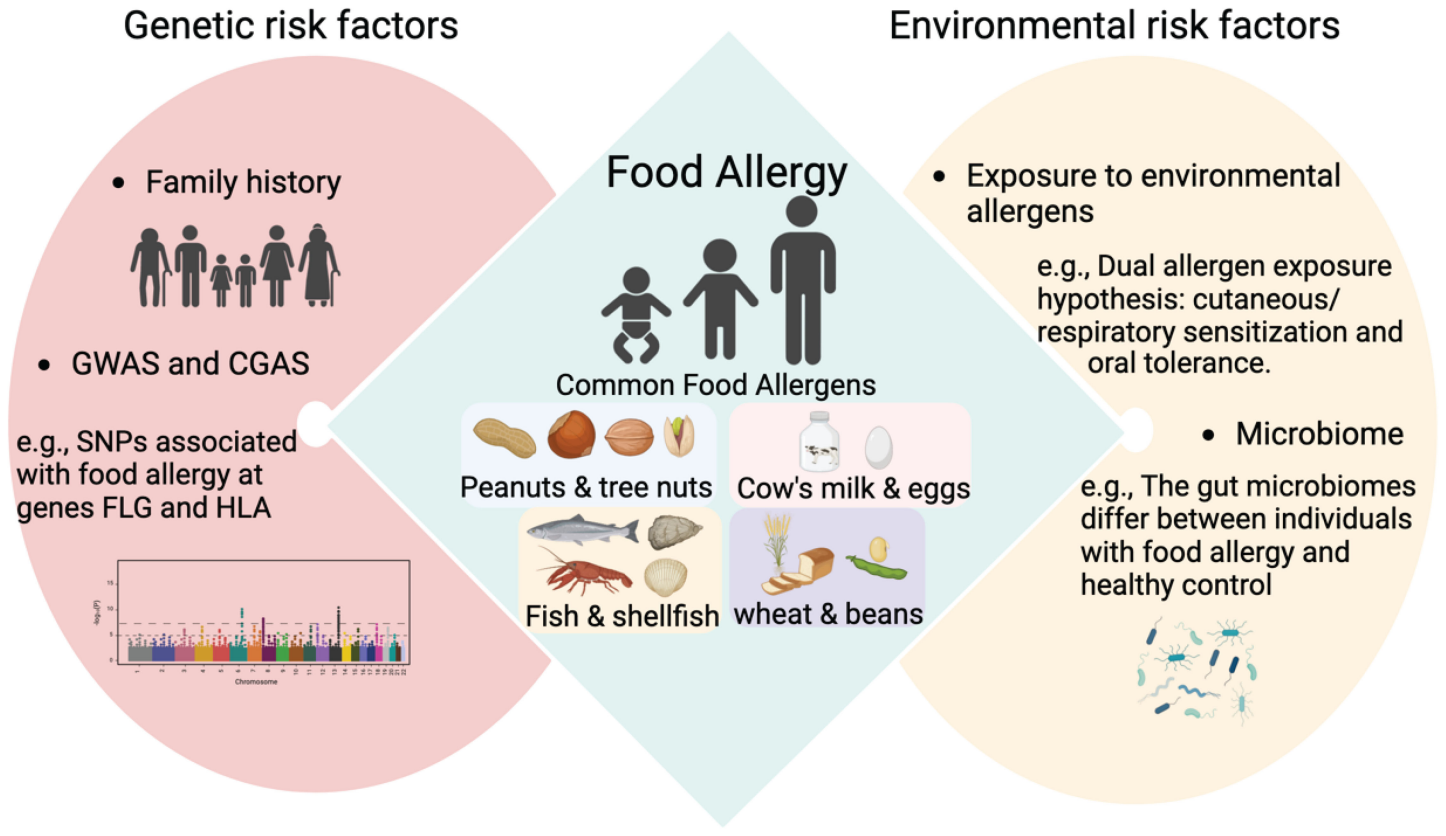
Main risk factors associated with FA. GWAS, genome-wide association study; CGAS, candidate gene association studies; SNPs, Single Nucleotide Polymorphisms; FLG, filaggrin; HLA, human leukocyte antigen.

The etiology of FA is complex and mediated by both genetic and environmental factors (**Figure 3**) (36, 37). A few twin and familial studies have been conducted and they have demonstrated the significant role of genetic factors in risk of developing FA (37). A twin study in the United States, involving 14 pairs of monozygotic (MZ) twins and 44 pairs of dizygotic (DZ) demonstrated that the concordance of peanut allergy is much higher among MZ twins (64.3%) compared to DZ twins (39.2%) (38). In another twin study (39), a significantly higher concordance rate for peanut allergy was found among MZ twins than among DZ twins, strengthening the evidence of heritability of peanut allergy. In addition, this study, for the first time, showed a similar genetic effect among patients allergic to pistachio, walnut, sesame, and fish (39).

A single-center study involving 5,276 infants from the HealthNuts study cohort in Australia investigated the impact of family history of allergic disease as an indirect measure of genetic risk for developing FA in the first year of life (40). OFC for egg, peanut, and sesame were used to diagnose FA in this study (40). This study revealed that compared to those with no family history of allergic disease, having one immediate family member with a history of any allergic disease modestly increased the risk (odds ratio [OR] 1.4, 95% CI 1.1–1.7) of developing FA and having two or more allergic family members was more strongly predictive of FA (OR 1.8, 95% CI 1.5–2.3) (40).

Recent reviews have summarized FA-associated genetic loci and genes by genome-wide association study (GWAS) and candidate gene association studies (CGAS) (36, 41). Specifically, the genetic variations in the genes filaggrin (FLG) (26, 42, 43)and human leukocyte antigen (HLA) (44–47) have reproducibly been found to be associated with FA. It was estimated that FLG gene mutations represent a risk factor for the onset of severe reactions from FA (OR = 8.9; CI: 3.1-28.3). Atopic children carrying filaggrin mutations represent a high-risk population due to their predisposition to develop severe FA reactions, such as anaphylaxis (26).

Environmental factors have been demonstrated as key determinants for FA risks (48–50). Although the concordance rate of FA in MZ twins is higher compared to that in DZ twins as shown in the twin study (38), among 14 pairs of MZ twins, 5 of them were discordant for peanut allergy. In other words, the concordance rate among MZ twins is less than 100%, suggesting the potential influence of environmental factors on the development of FAs. Furthermore, the familial study (40) demonstrated that the risk for FA in infants with two allergic family members compared to those with no family members with allergies was lower in children with both parents born in Australia than in those with both parents born in Asia. The overall lower rates of reported FA among parents born in East Asia, but higher rates among their infants, again suggests interactions with environmental factors.

A study measured environmental peanut exposure (EPE) using peanut protein levels in household dust and demonstrated an exposure-response relationship between EPE and peanut SPT or peanut-specific IgE (51). Furthermore, this study showed that the effect of EPE on peanut SPT and peanut specific IgE was augmented in children with a history of AD, strengthening the dual allergen exposure hypothesis (51). Expansion of TSLP-elicited basophil locally in the skin, Th2 cytokine production from T cells, proliferation of intestinal mast cell, and IgE-mediated anaphylaxis can be induced in mice sensitized through the epicutaneous route (22, 52). In addition to the skin, the dual allergen exposure hypothesis has been proposed to extend to the airways (53). A recent study demonstrated that patients with FAs had higher levels of serum IgE specific to house dust mites (HDM), one of risk factors for airway diseases, compared to healthy control (54). This study also used a mouse model to show that exposure to HDM contributed to the development of ovalbumin (OVA)-induced intestinal allergy, as evidenced by increased production of Th2 cytokines, and IgE antibody levels, as well as induced intestinal barrier dysfunction (54).

There is increasing evidence suggesting an association between altered microbiota (diversity and composition), and the pathogenesis of FA (55). Modern lifestyles with the overuse of antibiotics and hand sanitizers, a shift from rural to urban living, sterile indoor environments, and an increase in births by caesarian section have all altered our microbiome, which have health implications. A recent study on twin pairs revealed a bacterial signature of 64 operational taxonomic units (OTUs) that distinguished healthy from allergic twins (56). The OTUs enriched in healthy twins mainly belonged to the Clostridia class (56). This study also demonstrated that the enrichment of diacylglycerol in healthy twins may act as a potential measurable fecal biomarker of health (56). Phascolarctobacterium faecium and Ruminococcus bromii have been shown to be significantly associated with healthy twins, suggesting new possibilities for the development of live microbiome-modulating biotherapeutics (56). Another recent study involving participants in the CHILD birth cohort (n = 1115) in Canada, demonstrated that delayed infant microbiota maturation, as determined by profiling stool samples collected at ages 3 months and 1 year, was shared amongst various types (AD, asthma, FA, and AR) of allergy diagnosis at 5-years compared to those with no history of allergic sensitization (57). This finding suggests that the maturation of microbiota may serve as a key indicator to predict and prevent allergic diseases and underscores the crucial role of gut microbiota in early life in the development of allergies (57).

Epidemiological studies have suggested that air pollution is a major risk factor contributing to the global health burden, particularly respiratory diseases such as asthma and AR, however, studies on the role of air pollution on FA is limited (58, 59). The common sources of air pollution include household combustion devices, emissions from motor vehicles, industrial facilities, and forest fires (60). Air pollution is a complex mixture containing both particles and gases. Air pollutants that have the most compelling evidence for public health concerns include particulate matter (PM), carbon monoxide (CO), ozone (O_3_), nitrogen dioxide (NO_2_), and sulfur dioxide (SO_2_) (61). A recent study involving 2598 children aged 3–6 years in China demonstrated that the prevalence of FA was associated with prenatal and postnatal exposure to outdoor air pollution, particularly the traffic-related air pollutant NO_2_ (62). However, the role of air pollution in FA development remains unclear as results have not been consistent across studies (49).

## Prevention of food allergies

While prevention of food sensitizations and FAs and building tolerance to food allergens is the ultimate goal, preventing allergic reactions and improving quality of life in those with FAs is also a high priority. Studies have evaluated timing of allergen introduction to build tolerance, changes to maternal and infant diets (vitamin D, omega 3 fatty acids, prebiotics, probiotics, diet diversity), breastfeeding, and the role of emollients as potential means to prevent development of FAs. Prevention of allergic reactions in those with FA includes education and awareness of hidden allergens and introduction and implementation of labeling laws to prevent accidental ingestion.

### Timing of allergen introduction

Guidelines for the timing of allergen introduction during pregnancy, lactation, and infancy to prevent FAs has undergone major shifts in their recommendations in recent years. Earlier guidelines recommended avoidance of common food allergens during pregnancy and lactation and delaying the introduction of allergenic foods in infants to between 1-3 years of age (63–66). In infants, two pivotal studies on infant feedings led to the re-evaluation of these guidelines. In the Learning Early About Peanut (LEAP) trial (67), infants at high risk for peanut allergy (severe eczema and or egg allergy) but with peanut SPT wheals of 4 mm or less were randomized to consume or avoid peanut to age 5 years. The results of the study indicated that peanut allergy was significantly higher in children who avoided peanuts. The Enquiring About Tolerance (EAT) (68) trial introduced six common food allergens (cow’s milk, hen’s egg, peanut, sesame, cod fish, and wheat) after exclusive breastfeeding and showed the benefits with cooked egg and peanut. Based on these results, FA prevention guidelines reversed previous recommendations of allergen avoidance and instead recommended early introduction of allergenic foods. While there is a consensus that allergen introductions should start early, the allergens that should be introduced and the timing of introduction vary between international guidelines, and these are tabulated in the paper by Sampath et al. (8) The evidence for prevention of FA for peanut and egg is high; however, while evidence of benefit of other allergens have not been established, there is no evidence of harm.

While most guidelines generally recommend exclusive breastfeeding for the first 4-6 month before allergen introduction, there is no evidence that breastfeeding prevents development of allergic disease, although it is known that food allergens cross the placental barrier (69) and is found in breast milk (70). There is also no strong evidence of use of milk substitutes in infancy for prevention of allergic diseases (8). The EAACI guidelines also recommend against the use of regular cow’s milk formula in the first week of life (71).

More recent guidelines now recommend consumption of a healthy and diverse diet during pregnancy or breastfeeding with consumption of allergenic foods in normal amounts and discourage avoidance diets due to its potential to adversely affect nutrient quality (72). However, debate whether introduction of allergenic foods during pregnancy and lactation prevents allergic sensitization continues. A study found that infants who were introduced to peanut before 12 months of age after cessation of breastfeeding had a 66% reduced risk of sensitization at 5 years compared to those who were not. Further, they found that if mothers introduced peanut early while they were breastfeeding and were also regularly consuming peanut themselves, this risk was further reduced. These results suggest that maternal peanut consumption in addition to breastfeeding at the time of peanut introduction during infancy may help to decrease the risk of peanut sensitization (73).

### Diet diversity

Diet diversity during infancy has also been hypothesized to prevent FA potentially by modulating the gut microbiota via exposure to a variety of foods and promoting immune tolerance (74, 75). Venter et al. evaluated the effect of introducing several foods to infants and increasing diet diversity during the first year of life on the development of allergic diseases. Diet diversity was defined as either (1) minimum diet diversity (World health organization classification), (2) food diversity, (3) fruit and vegetable diversity, and (4) food allergen diversity. The study found that, over the first ten years of life, the introduction of each additional food at 6 and 12 months of age reduced the odds of developing FA by 10.8% and 33.2% respectively (76). A systematic review suggests that diet diversity in infancy may be associated with reduced allergy outcomes (including FA) (77). In a 1 year pilot study, infants between 4-6 were fed multiple allergens. The results demonstrate that infants were able to tolerate the early introduction of multiple allergenic foods. Further, infants consuming multiple allergens showed trends for improvement in food challenge reactivity and plasma biomarkers (78). A systematic review evaluated diet diversity and allergy outcomes in infants and children and found no clear association and concluded that further studies were needed (77).

### Vitamin D and omega-3 fatty acids

Some studies have found an association between omega-3 fatty acids and vitamin D and decreased risk of FA, but these results have not been consistent. A meta-analysis found a linear dose dependent relationship between omega-3 supplementation during pregnancy and lactation and decreased risk of infant egg sensitization risk during early life. This decreased FA risk was not observed on intake of omega-3 polyunsaturated fatty acid during childhood (79). A systematic review and meta-analyses of 43 studies found no evidence of an association between maternal antenatal or infant vitamin D levels or dietary intake and the development of FA or eczema in offspring (80). However, another systematic review and meta-analysis found that decreased maternal vitamin D levels and infant vitamin D insufficiency increased the incidence of FA s, particularly in the second year of life (81). Another literature review evaluated studies on vitamin D and omega-3 fatty acid supplementation and concluded that these supplements may be beneficial for prevention of FA in some populations and that further research that takes into consideration the predisposition to allergy, amount of sunlight exposure, and the overall nutritional composition of the maternal diet may shed further information (82). A recent study demonstrated that regulatory CD4 T cells (Tregs) were statistically increased in infants with IgE-mediated peanut or egg allergies who had adequate vitamin D levels compared to those with inadequate vitamin D levels (83). This suggests that vitamin D may enhance circulating levels of Tregs in infants with FA s (83).

### Probiotics

Probiotics have been suggested as potentially reducing risk of FA by altering gut microflora or providing nutrients for gut microflora, respectively (72, 84). Increased permeability of the gut barrier has been shown to increase translocation of allergenic molecules leading to Th2 inflammatory immune responses. Probiotics are thought to enhance gut barrier function and promote the restoration of healthy gut microbiota (85). A systematic review and meta-analysis of 37 studies found that probiotics supplementation during pregnancy and infancy reduced the risk of total FA, cow-milk allergy, and egg allergy, infancy-only supplementation lowered cow-milk allergy risk, and pregnancy-only had no discernible effect (86). The role of probiotics in preventing FAs are still under investigation. Currently guidelines do not recommend their use for prevention of FA (8). Probiotics are also being evaluated as treatment for those with FA and these are discussed in a later section.

### Emollients

Maintaining a healthy epithelial barrier to prevent FAs has been explored by numerous studies for the prevention of FA. However, results are currently mixed. The PEBBLES pilot study in 2018 was the first to evaluate the role of emollients in preventing food sensitization in addition to AD and showed trends towards decreased risk of food sensitization at 6 and 12 months of age in addition to a trend towards decreased risk of AD at 12 months. Per protocol analyses which only including infants who received study treatment for  5 days/week or fewer revealed a significant reduction in food sensitization at 12 months in the treatment group (87). Two additional large randomized controlled studies in 2020, the BEEP and the PreventADALL found no associations between use of emollients and FA rates. The PreventADALL study was the first large, population based, randomized clinical trial investigating the combination of early introduction of food allergens and regular emollients to prevent atopic dermatitis and found no differences between control and emollient groups in decreasing rates of atopic disease (88); similarly the BEEP study found no differences in rate of FA or food sensitization (milk, egg, or peanuts) at 2 years (89). One potential reason for the lack of association between emollient and FA risk is that these studies used a paraffin-, alcohol-, or petroleum-based emollient rather than a trilipid emollient. These emollients have a 3:1:1 ratio of ceramides, cholesterol and free fatty acids. These mimick the skin’s pH and lipid composition. A study using trilipids as emollient is currently in phase 2 clinical trials (Clinicaltrials.gov, NCT03742414).

### Allergen avoidance

In a cross-sectional survey 558 respondents with FA (children and adolescents), 73% reported an accidental ingestion since diagnosis (90). In a web-based survey in Japan, of 1,141 valid responses (5-15 years of age), 67.4% participants had a history of anaphylaxis, and 54.5% experienced accidental ingestion (91). To combat accidental ingestions, avoidance measures such as manufacturing regulations regarding labeling and advisory warnings, restaurant staff education and cooking methods to avoid allergen exposures, providing such as booking a room with a separate kitchen area, emergency plans for school (mealtimes, field trips, substitute teachers, bus travel), vigilance by the child and caregiver, experimentation and exposures to different food groups, and offering proper nutritional and medical monitoring would assist with improving quality of those with FA (92–94). There is ongoing work to advance food allergen labeling laws around the globe. However, there is still many gaps in labeling laws and its enforcement. Avoiding accidental ingestions and anaphylaxis currently requires the vigilance and education of the individual, who must familiarize themselves with labeling laws (95).

## Diagnosis of FA

FA is commonly diagnosed by a combination of clinical history and measurements of specific IgE (either by SPT or serum IgE levels) (8, 19, 96, 97). However, double-blind placebo controlled oral food challenge (OFC) is still considered the gold standard diagnostic test for FA (98). However, this test is time consuming and involves the risk of anaphylaxis, which can be life-threatening and therefore used only in specialized clinicals (96, 99). Other biomarkers of potential use for FA diagnosis and prognosis include basophil activation test (BAT), mast cell activation (MAT), and component-resolved diagnostics (CRD). Other tests such as IgG4, IgE glycosylation, T and B cell assays, microbiome analysis, and plasma cytokines are also being explored (100).

### SPT and IgE

SPT for FA assessment involves introducing a small amount of the food allergen into the epidermis. The test site is examined approximately 15-20 minutes later for the presence of a wheal, which occurs due to the release of histamine from mast cells (101). SPT is typically considered positive if the wheal formed around the test site measures at least 3 mm greater in diameter than the negative control site (96). Positive results of sIgE level have been traditionally considered an sIgE ≥0.35 kU/L (96). However, these cutoffs have poor specificity to clinical FA, as about half of sensitized individuals able to tolerate the food without reaction (102). As higher values are correlated with a higher risk of reaction, a positive predictive value of 95% or greater indicating a very high probability of an allergic reaction to the suspected allergen has been determined for certain allergens (10). It should be noted that severity of allergic reactions to foods cannot be predicted by the level of sIgE or the size of the SPT wheal (103). Hill et al (104)demonstrated that SPT wheal diameters of ≥8 mm for milk, ≥7 mm for egg, and ≥8 mm for peanut corresponded to a 100% positive predictive value (PPV) for the diagnosis of FA. They also found that the diagnostic accuracy of SPT and IgE antibody levels was similar for cow milk, but SPT was more sensitive in diagnosing allergy to egg and peanut (104). A recent study demonstrated SPT thresholds ranging from 4.5 mm for wheat to 14.5 mm for egg in predicting a positive OFC, with Area Under the Curve (AUC) values ranging from 0.52 to 0.90; sIgE thresholds ranged from 1.2 kU/L for cashew to 52.2 kU/L for wheat in predicting a positive OFC, with AUCs ranging from 0.59 to 0.92 (105).

### BAT and MAT

Basophil activation tests (BATs) have recently been applied in the diagnosis of cow’s milk, egg, and peanut allergies (106–108). BAT has a high sensitivity and high specificity to diagnose FA. It is a flow cytometry-based assay that examines the capacity of IgE to activate basophils upon exposure to the allergen through IgE cross-linking (106, 108). The major activation markers expressed on basophil cell membrane include CD63 and CD203c (109). A study demonstrated that BAT in peanut-allergic children exhibited a peanut dose-dependent upregulation of CD63 and CD203c (109). This study also identified the diagnostic cutoff values for %CD63^+^ basophils at 100 ng/mL and mean %CD63^+^ basophils at 10 and 100 ng/mL of peanut extract, and these cutoff values showed 97% accuracy, 95% positive predictive value, and 98% negative predictive value (110). The BAT has also been demonstrated to have superior accuracy compared to tests for IgE sensitization (sIgE/SPT) to peanut, Arah2 or cow’s milk (110, 111). A study involving participants from the Markers of Nut Allergy Study (MONAS) cohort, ranging in age from 0.5-17 years with confirmed peanut and/or tree nut (almond, cashew, hazelnut, pistachio, walnut) allergy or sensitization, showed that the BAT has the potential to predict allergic clinical status to peanut and tree nuts in multi-nut sensitized children (112). These findings suggest that BAT may alleviate the need for OFCs in diagnosis of FA in certain patients (112). A drawback of BAT is that it requires whole fresh blood within 24 hours of sampling. Additionally, in 10-15% of individuals, the BAT results are uninterpretable as the basophils do not respond to IgE-mediated stimulants (113). These limitations hinder wider applicability of BAT.

Mast cell activation tests (MAT) is similar to BAT but uses plasma or serum to sensitize mast cells lines rather than whole blood (114). Expression of activation markers are measured on stimulation with allergen. The MAT has similar specificity in the diagnosis of PA but lower sensitivity (115). BAT and MAT are still used in laboratory settings and requires appropriate standardization and validation before widespread use in a clinical setting.

### Component resolved diagnostics

Greater availability of highly purified native or recombinant proteins have now enabled use of single allergens for component-resolved diagnosis of FA. These can provide higher diagnostic accuracy and enable predictions of severe reactions (116). Understanding structural similarities between allergens can also assist in understanding cross-sensitization. A study involving 150 children age 3.5 to 18 years in the Netherlands found that specific IgE to Ara h 2 was a strong predictor of peanut allergy and showed high discriminative capacity (117). Similarly, a study of 48 peanut-allergic children in France found that high Ara h 2 sIgE titers could predict the risk of anaphylaxis (118). Other allergenic components associated with severe reaction include Gly m 4 (soy), Omega-5 gliadin (wheat, exercise induced anaphylaxis), and Cor a 9 and Cor a 14 (hazelnut). Jug r 6 (walnut) has been shown to cross react with hazelnut, sesame and pistachio (10).

## Treatments

In the last few decades, there has been significant progress in our understanding of the mechanisms underlying immune tolerance and allergy for foods, which have assisted in the development of novel therapies and drugs (20, 119–122). The ultimate goal of treatment is a cure, to induce of a state of permanent tolerance, via immune modulation. Current treatments, however, fall short of a cure. Effectiveness of immunotherapy for FA has been known since the early 1900s, but it was rarely attempted due to concerns of severe allergic reaction. Only in the last few decades has there been renewed interest in its use for FA with significant advances in immunotherapy protocols, including those using purified or altered allergens or with adjunctive therapy (123, 124). Palforzia is an immunotherapy drug, which was the first drug approved for the treatment of FA, specifically for peanut allergy (125). This was followed by omalizumab, which was approved by the US FDA in 2024. In addition, there are a number of other biologics, vaccines, and other novel therapeutics being evaluated in advanced clinical trials (126–128). Here, we review the current status of FA treatments and the limitations and challenges ahead (overview provided in **Table 1**).

**Table 1:** Treatments for FA and other atopic diseases.

Class	Target Pathways or mode of delivery	Drug/Platform	Comments	FDA Approval Status or Clinical trial status
Immunotherapy	Oral immunotherapy: As monotherapy or as conjunctive treatment with biologics, probiotics, or with a Chinese Herbal formula.	Palforzia (characterized peanut formulation for those with peanut allergen). Appropriate whole foods are also used as therapy for peanut and other FA s.	OIT is associated with high rates of desensitization and patients can consume high allergen levels after therapy. Concerns include high rates of allergic events, including severe reactions and anaphylaxis. Durability of treatment generally requires continued ingestion of allergens.	Palforzi is approved for peanut allergy. Other OIT conjunctive treatments are in clinical trials.
	Epicutaneous route of delivery. Dried allergen extracts administered through dermal patches	Viaskin	Desensitization occurs with EPIT but at much lower levels than with OIT. No major safety issues are indicated except for localized reactions at site of application.	In phase 3 clinical trials
	Subcutaneous route of delivery	NA	Highly efficacious but high rates of anaphylactic events have limited clinical trials. More recent studies use recombinant or chemically modified forms of allergens.	In phase 2 clinical trials using chemically modified forms.
	Sublingual mode of delivery Allergens delivered via tablets or drops under tongue	NA	Less effective than OIT as dose is limited by amount of allergen deliverable. Side effects are minimal, typically limited to oropharyngeal itching.	In phase 2 clinical trials
Biologics	Anti-IgE	Omalizumab	Significantly increases tolerated dose of multiple foods as monotherapy; decreases adverse events and time to sensitization as OIT adjunctive.	Approved for FA. Also used in conjunction with OIT for single or multiple FA s.
		Ligelizumab (QGE031)	Ligelizumab’s binding affinity for free IgE is approximately 88-fold higher than omalizumab.	In phase 3 clinical trials for FA
		UB-221	UB-221 binds IgE with an 8-fold higher affinity than omalizumab.	No clinical trials for FA but has been evaluated for phase 2 chronic spontaneous urticaria.
	Anti-IL-25 antibodies	*XKH001, Brodalumab (AMG 827)*	Il-25, IL-33, and TSLP are together called Alarmins: In mice, significant increases in tolerance and strong inhibition of FA development when used together.	In trial for asthma (*Brodalumab). XKH001 in early clinical trials for assessing safety in health volunteers.*
	Anti-TSLP antibodies	Tezepelumab		Approved for allergic asthma.
	Anti-IL-33 antibodies	*Itepekimab, Etokimab* *(ANB020)*		In phase 2 clinical trial for FA (*Etokimab). Itepekimab in clinical trials for other diseases.*
	IL-4R (receptor common to both IL-4 and IL-13)	Dupilumab	IL-4, Il-5, and IL-13 are Th2 cytokines and antibodies targeted to these cytokines or their receptors have been approved for asthma or other atopic diseases.	In phase 2 clinical trials for FA as monotherapy or in conjunction with OIT (with and without omalizumab)
	Anti IL-5 or IL-5R	Reslizumab and Mepolizumab (Anti IL-5), Benralizumab (Anti Il-5R),		Approved for asthma (Benralizumab and Reslizumab). Mepolizumab approved for Chronic rhinosinusitis with nasal polyps
	Anti -IL-13	Lebrikizumab, Tralokinumab		Both approved for atopic dermatitis. In clinical trials for other atopic diseases.
JAK-1/NTK inhibitors	Encapsulate allergens, protect from degradation and enable target therapy	Abrocitinib	*In vitro* study demonstrated attenuation of Th2 responses on peanut exposure in blood obtained from peanut allergic individuals.	In phase 1 clinical trial for FA.
Bruton’s tyrosine kinase inhibitor		Acalabrutinib	Bruton’s tyrosine kinase (BTK) inhibitors block signaling downstream of the FceRI.	In phase 2 clinical trials for FA. Approved for Chronic lymphocytic leukemia,
Vaccines		ASP0892 (DNA vaccine) PVX108 (peptide vaccine)	Safe and well-tolerated in peanut allergies and demonstrated increased tolerance Further investigation needed to ascertain efficacy	In phase 1 clinical trials (ASP0892) In phase 2 clinical trials (PVX108)

FA, food allergy

### Immunotherapy

Immunotherapy has been known since over 100 years. The first study on immunotherapy for allergy was published in 1911 for allergic rhinitis (129). Since then, while rare reports of successful immunotherapy for FA have been reported in the early part of the last century, it was largely ignored till the 1990s due to safety concerns. In immunotherapy, patients are exposed to small but increasing doses of the allergen, slowly building immune tolerance to the allergen and increasing a patient’s allergic threshold, which is the maximum amount of an allergenic food that a patient can tolerate without producing any adverse reactions. While the immune modulatory effects of immunotherapy are well established (121, 130, 131), immunotherapy is associated with a number of limitations and challenges, which has impeded widespread use. These include (1) lack of standardized protocol, (2) lack of standardized allergen, (3) risk of severe or even fatal anaphylactic reactions, (4) lengthy treatment (often years) and frequent clinic visits, and (5) impermanence of desensitization.

In most patients, continued ingestion of the allergen after successful immunotherapy is required to maintain desensitization (132). If treatment is discontinued, the patient most often regains sensitization to the allergen. The period of desensitization that occurs after immunotherapy is termed remission (also called sustained unresponsiveness in earlier studies). There is no established standard for the period of allergen avoidance, and these vary between 2 week and 6 months between studies (133), making comparisons of remission between studies difficult. Here we discuss the various modes of administration of immunotherapy and their current status and their limitations and challenges.

Three main routes of administration of allergens, which differ in ease of use, efficacy, and safety have been used for food allergen immunotherapy. These include oral immunotherapy (OIT), epicutaneous immunotherapy (EPIT), and sublingual immunotherapy (SLIT). Subcutaneous immunotherapy (SCIT) has also been attempted for FA and shown to be effective, but due to high risk of anaphylaxis in earlier studies and one fatality, it took a backseat to other forms of administration. However, there has been some renewed interest in this form of administration in recent years (134). For FA, OIT is the most common form of immunotherapy (primarily for peanut, egg, or milk allergies) (135).

### Oral immunotherapy

One of the earliest report of successful OIT for FA was in 1908, when it was reported that ingestion of egg in increasing doses led to desensitization to egg (136). In 1930, a study by Freeman suggested that immunotherapy for fish allergy was effective in desensitizing the individual to the allergen (137). However, it was only in 1998, that one of the first controlled OIT study for FA was reported (138). Although significant advances have been made since then, there is still no standardized protocol for OIT, and treatments vary with respect to type and dose of allergen used, the frequency and length of treatment, adjuvants or pretreatments used (if any), and goals of treatment outcome. Some studies aim to increase reaction thresholds to amounts that enable individuals to ingest allergenic foods to near normal dietary levels, while others set reaction thresholds at lower amounts but adequate to prevent allergic reaction on accidental ingestion (139, 140).

OIT generally consists of at least 3 phases: a screening phase, a build-up phase, and a maintenance phase (141). The build-up or dose escalation phase can last 6-8 months or even longer. At the end of the maintenance phase, which could last a few weeks to years, an oral food challenge is conducted to assess desensitization. In some clinical trials, a fourth phase called the tolerance phase to determine remission is included (142). Due to a lack of standardized food allergens for most FA s, protocols use commercial whole food flours. These food flours are still highly variable in allergic and non-allergenic protein content and protein type. The only standardized allergen that has been developed and approved by the US food and Drug Administration (FDA) and European Medicines Agency (EMA) is Palforzia. It is a highly characterized pharmaceutical-grade, peanut OIT formulation that is designed to provide consistent dosing of peanut allergens. It is standardized to Ara h1, h2, and h6, the three major allergenic proteins in peanuts. It is to be used as an OIT treatment for the mitigation of allergic reactions, including anaphylaxis, that may occur with accidental exposure to peanut for those aged 4-17 with peanut allergy (143). For patients with multiple FA s and those with FAs other than peanut, there are currently no approved drugs. It is estimated that over 200 foods are associated with FA s of which eight are considered as major allergens accounting for 90% of the FA reactions (144). Results of a large, nationally representative sample of US children and adults found that 40% and 48% had multi-FA, respectively (14). For these individuals, whole food flours, which are not FDA regulated, are used for OIT at specialized allergy centers. Simultaneous OIT for multiple allergens with food floors have also been shown to be feasible and are performed with the use of adjunctive treatment to improve safety and decrease time to desensitization (145).

OIT has been shown to be highly efficacious. Systematic reviews and meta-analyses of peanut, egg, and milk allergy indicate OIT is effective in desensitizing individuals to their allergens, with high rates of success at around 80-85% (121, 146–148). In two separate phase three clinical trials, it was shown that 50.3% to 58.3% of those treated with Palforzia could tolerate a 1,000 mg peanut protein challenge (125, 143). Studies have also measured durability of desensitization. A study by Chinthrajah et al., found that in those achieving desensitization to peanut allergy after 2 years of OIT, remission was observed in 35% and 13% at 3 months and 1 year, respectively (149). In another study of children with egg allergy, 27.5% achieved remission at 2 years after end of OIT (150).

Safety of OIT is a major concern and a meta-analysis and systematic review of randomized controlled studies found allergic events were increased with intervention, and risk of adrenaline use increased for peanut, egg, and milk (133). Rates of withdrawal vary between 10%-20%, but some studies have reported rates as high as 36%, primarily due to adverse events (141, 151). Another systematic review found significantly higher rates of overall allergic reactions among patients receiving OIT as compared to avoidance management (146). In those treated with Palforzia, 18.2-25.3% of patients experienced moderate symptoms and 4.5-5.1% of patients experienced severe symptoms (125, 143). Most adverse events with OIT are mild, typically involving oropharyngeal pruritus and mild transient gastrointestinal symptoms, however, on occasion, severe reactions, such as wheezing, vomiting, urticaria, and angioedema are reported (152). Severe cases of anaphylaxis, although rare, have been documented to occur to doses of allergen previously tolerated, usually triggered by cofactors such as infection, exercise, anxiety, or allergen co-exposure (141). Another concern is Eosinophilic eosinophilia (EoE) (153, 154). Estimates of the incidence of EoE during OIT vary from 2.7% to as high as 14%, depending on the method used to determine EoE and the type of allergen (153, 155). However, symptoms generally resolve upon discontinuation of OIT (156). In one study, a third of patients reported mild symptoms suggestive of EoE before OIT (155). Adverse events are dose related and therefore studies have evaluated the impact of lower allergen dose on efficacy. Vickery et al. found that in infants with peanut allergy, a 300 mg maintenance dose with peanut protein has similar efficacy and improved safety profile as a 3000 mg dose (157). A low dose of 200 mg sesame protein was found to be safe and effective in desensitizing preschoolers with sesame allergy (158), a dose much lower than previous doses of 1200 mg, a dose that is not feasible or practical for preschoolers (158). The maintenance dose for Palforzia is low, at 300 mg peanut protein (159). Current studies are therefore using much lower doses with the goal of increasing allergen threshold to that which protects from severe reaction on accidental consumption. Other studies are using adjunctives, such as anti-IgE, to decrease adverse effects and lower time to desensitization. These are discussed under the section on OIT with biologics and adjunctives.

### Epicutaneous immunotherapy

EPIT is also a promising approach for FA immunotherapy. The first pilot study of EPIT for FA was conducted in 2010 in children with milk allergy. It showed a trend towards clinical efficacy and was well tolerated (160). Currently, the Viaskin platform is the most advanced technology for EPIT. It is a proprietary delivery system consisting of an adhesive dermal patch containing dried allergen extracts. The dried allergens are solubilized on contact with the moisture on the skin and is delivered into the epidermis. Patients start with a small initial dose and wear the patch for increasing periods of time until a maintenance dose is reached, after which each patch is worn 24 hours and replaced daily. Dosing is lower than in OIT and are in the microgram range rather than in milligram or gram amounts. EPIT is safer than OIT with localized reactions. However, OIT can increase allergen threshold to much higher amounts than EPIT. In a phase 3 multicenter, double-blind, randomized, placebo-controlled trial study for peanut allergy maintenance dosing was 250 μg of protein. The study found that EPIT for 12 months was superior to placebo in desensitizing children 1 to 3 years of age to peanuts and increasing the threshold dose that triggered allergic symptoms. Serious treatment-related adverse events and anaphylaxis occurred in 0.4% and 1.6%, respectively, in the intervention group and none in the placebo group (161). Other approaches to EPIT that are being developed are allergen-coated microneedles, application of allergen on the skin (either by pretreatment by tape stripping, abrasion or laser-mediated microperforation) (162). A complete list of clinical trials on EPIT using Viaskin technology can be found in the review by Herve et al. (162) A systematic review and meta-analysis found that immune tolerance to food in increased after EPIT. However, the study also found that it also significantly increases mild-to-moderate anaphylaxis (163).

### Subcutaneous immunotherapy

SCIT is commonly used for treatment of allergic rhinitis. SCIT, while effective for FAs is associated with unacceptably high rates of adverse systemic reactions for FA and therefore there are only a few studies on SCIT for FA (164). A small pilot study of 6 patients, however, suggests that patients with pollen FA may find relief with SCIT for aeroallergens. The study found that in patients with pollen FA associated with soybean allergy, median ingestible amount of soy milk was increased from 1.5 mL to 150mL 1 year after initiating Birch SCIT; however, incidence of systemic reactions was 67% in the rapid escalation phase, indicating that the protocol for Birch SCIT needs further improvement (165).

Research on reducing allergenicity and therefore risk of serious adverse events with SCIT is now being evaluated. Recombinant allergens or allergen extracts adsorbed on Aluminium hydroxide have been shown to have lower allergenicity. Animal studies using sera of fish-allergic patients found that use of recombinant hypoallergenic carp parvalbumin, Cyp c 1 adsorbed on aluminium hydroxide resulted in a 10- to 5,000-fold (1,000-fold on average) reduction in allergenic activity with retained immunogenicity with the potency to stimulate human peripheral blood mononuclear cells (PBMCs) (166). In a phase 1 SCIT study, chemically modified, aluminium hydroxide adsorbed on peanut extract (HAL-MPE1) was found to be safe and tolerable with immunological changes in peanut allergic patients (167).

Nutritional interventions can also potentially improve safety of SCIT. In a study in mice, supplementation of normal diets with non-digestible oligosaccharides were found to reduce anaphylaxis caused by a single peanut extract SCIT dose. This effect appears to be due to a direct effect on mast cells, since the non-digestable oligosaccarides reduced mast cell degranulation (168).

### Sublingual immunotherapy

SLIT is another alternative to OIT which has proven to be safe and effective (169, 170). It involves placement of allergen either as a tablet or drops under the tongue daily to achieve allergen-specific desensitization. SLIT has been studied in the treatment of kiwi, hazelnut, peach, apple, milk and peanut allergies. In a phase 2 study, where children with peanut allergy underwent SLIT (2 mg/d peanut protein for up to 5 years), 25% were desensitized with 20.8% achieving remission (171). In a randomized study of peanut allergic children who either received 4 mg peanut SLIT or placebo, desensitization was assessed by oral food challenge after 36 months of treatment. Participants desensitized to at least 443 mg peanut protein discontinued therapy for 3 months and then underwent food challenge to assess for remission. Desensitization and remission was observed in 1- to 4-year-old children, with improved outcomes seen with younger age at initiation. Further, changes in SPT, IgG_4_, and IgG_4_/IgE ratio were seen in peanut SLIT but not placebo participants (172). In an open-label study of the efficacy, safety, and durability of peanut SLIT in peanut-allergic children, the mean successfully consumed dose increased from 48 to 2723 mg of peanut protein after 48 months of treatment with 70% achieving clinically significant desensitization (> 800 mg) and 36% achieving full desensitization (5000 mg) (173).

SLIT has a better safety profile than OIT, but OIT is more effective than SLIT in inducing desensitization. SLIT efficacy is limited by the allergen concentration and the volume of liquid that can be administered. Side effects are minimal typically limited to oropharyngeal itching and occurs in less than 2-5% of doses (171, 174). There are no reports of severe anaphylactic reactions.

### Biologics

To date, a number of biologics have been approved for atopic diseases such as atopic dermatitis and allergic asthma but none for the treatment of FA. However, as common mechanistic pathways between FA and other atopic diseases exist, biologics effective for these other atopic diseases have potential for the treatment of FA, either as monotherapy or as adjunctive treatment with immunotherapy. These biologics target different effectors on the Th2 inflammatory pathway, either those cytokines initiating the cascade (epithelial cytokines) or those targeting IgE.

### Biologics targeting the epithelial cytokines (IL-25, IL-33, and TSLP)

The epithelial cytokines (alarmins) initiate the Th2 inflammatory pathway and it is hypothesized that biologics blocking the upstream mediators of the inflammatory pathway would be more effective than those blocking downstream mediators such as IgE. In a mouse model, injection of all three (anti IL-25, anti IL-33, and anti TSLP) monoclonal antibodies strongly inhibited FA development. However, injection of any of the above monoclonal antibodies singly could not suppress established FA and FA suppression was optimal only on treatment with all 3 anti-alarmins (175).

Of the three alarmins, only anti IL-33 has been evaluated in clinical trials for FA. Anti-TSLP (tezepelumab) is approved for allergic asthma; anti IL-25 antibodies *(*XKH001 and Brodalumab (AMG 827)) are in clinical trials for asthma (NCT05991661 and NCT01199289). A proof-of-concept 2a study in patients with peanut allergy, anti-IL-33 antibody was found to be efficacious as monotherapy in the treatment of peanut allergy in adults. The study found that a single dose of anti IL-33 resulted in a significantly increase with 73% of patients tolerating 275 mg of cumulative peanut protein at days 15 and 45 compared to 0% in the placebo group. IL-4, IL-5, IL-9, IL-13, and ST2 levels in CD4^+^T cells were reduced and peanut-specific IgE was reduced in active vs. placebo. There was also a trend in a reduction in atopy-related events in active vs. placebo groups during the study (176). Itepekimab is another IL-33 monoclonal antibody that is in clinical trials for chronic obstructive pulmonary disease (NCT04751487) but none for atopic diseases.

### Biologics targeting Th2 cytokines (IL-4, IL-5, and IL-13)

Key biologics targeting IL-4, IL-5, and IL-13 include dupilumab, mepolizumab, reslizumab, and benralizumab, lebrikizumab and tralokinumab. None of them are approved for FA but many have been approved for other atopic diseases. Of these, a number of clinical trials are ongoing with dupilumab. Dupilumab is directed against IL-4Rα, a receptor common to both IL-4 and IL-13. It is currently approved by both the Food and Drug Administration (FDA) for use as a biologic treatment in moderate-to-severe atopic dermatitis and asthma (177, 178). In a case report, a patient with severe atopic dermatitis receiving dupilumab became desensitized to two foods that the patient was previously allergic to (179). In a retrospective, case-control study, the total peanut IgE in the dupilumab group had median absolute IgE and percentage change of -0.98 kU/L and -3.67% per month, respectively, compared to the control group of 0.0 kU/L and 0.0% per month. Results of this small study suggests that dupilumab may cause a statistically significant decrease in food-specific IgE compared to controls (180). Dupilumab as monotherapy is currently in clinical trials for peanut allergy (NCT03793608), and as adjunctive treatment with OIT for milk allergy (NCT04148352) and peanut allergy (NCT03682770), and OIT plus omalizumab for patients with multiple FA s (NCT03679676).

Three monoclonal antibodies selectively inhibit IL-5: Mepolizumab, Reslizumab, Benralizumab. They inhibit the downstream action in eosinophils activation and recruitment (181). Mepolizumab is an anti-IL-5 antibody, indicated as an add-on maintenance treatment for patients 12 and older with severe eosinophilic asthma. Reslizumab is currently indicated as an add-on maintenance treatment for patients 18 years and older with severe asthma. Benralizumab binds to IL-5R and is indicated as an add-on maintenance treatment for patients 12 years and older with severe eosinophilic asthma.

Biologics targeting IL-13 include lebrikizumab and tralokinumab. Lebrikizumab is currently in phase 3 clinical trials for the treatment of atopic dermatitis (182). Tralokinumab is approved for the treatment of atopic dermatitis in patients 12 years and older.

### Biologics targeting IgE

In 2003, omalizumab, an anti-IgE antibody, was the first biologic that was approved for an atopic disease (asthma). It binds circulating IgE (but not IgE bound to mast cells and basophils) and blocks IgE binding to FcϵRI and prevents the activation and degranulation of mast cells and basophils and lowers risk of allergic reactions. It has been used as monotherapy (183) and as adjunctive treatment for single allergen and multiallergen OIT. It was first used as adjuvant therapy to OIT in 2011 in a pilot study of milk-allergic individuals. The protocol consisted of 9 weeks of omalizumab pretreatment, 7 weeks of omalizumab plus OIT, and 8 weeks of OIT alone as maintenance therapy. The study demonstrated that adjunctive omalizumab with OIT was efficacious, allowed for faster desensitization, and had a low frequency of adverse reactions (1.6% of doses), which were mild (184). A 2023 systematic review and meta-analysis found that omalizumab as monotherapy significantly increased the tolerated dose of multiple foods, increased the threshold of tolerated dose for milk, egg, wheat, and baked milk, improved quality of life, and reduced food-induced allergic reactions (185). In another study, omalizumab as adjunctive treatment to OIT, significantly increased the tolerated dose of multiple foods and was not associated with any major safety concerns. Increased in IgG4 levels were observed (185).

In a 36-week phase 2 trial of multi-allergic children, omalizumab increased the ability to pass an oral food challenge to at least 2-g food protein for 2 or more foods compared to placebo (83% vs 33%, respectively) and decreased time to maintenance. Omalizumab also improved the safety of multi-OIT by reducing the number of doses associated with AEs from 68% without omalizumab to 27% with omalizumab (186). Omalizumab was also found to increase the amount of peanut, tree nuts, egg, milk and wheat that children with multiple FA s could consume without an allergic reaction. After omalizumab treatment, around 67% of participants were able to consume at least 600 mg of peanut protein (approximately 2.5 peanuts) without a moderate or severe allergic reaction. In contrast, only around 7% of participants who received placebo were able to ingest similar amounts. Similar outcomes for egg, milk, wheat, cashew, walnut and hazelnut were observed (187).

Ligelizumab and UB-21 are other anti-IgE antibodies. Ligelizumab’s binding affinity for free IgE is approximately 88-fold higher than omalizumab. UB-221 also binds IgE with an 8-fold higher affinity than omalizumab and is superior in IgE neutralization and prevention of basophil degranulation. Ligelizumab is currently under investigation in patients with FA s (NCT05678959 and NCT04984876) (188). While UB-221 has not been evaluated in clinical trials for FA, a phase 1 study in patients with chronic spontaneous urticaria was associated with rapid reduction in serum free IgE and durable disease symptom score with reductions in weekly urticaria activity score (189).

### Immunotherapy with combination therapies

While immunotherapy has been shown to be efficacious for FA s, research to improve safety, permeance with desensitization, and reduction of treatment duration is ongoing. To address these concerns research into the use of adjunctive therapy with immunotherapy, such as antibodies targeting IgE and other cytokines associated with the Th2 inflammatory allergic cascade, Chinese herbal therapy, and probiotics are being evaluated.

### OIT in conjunction with biologics

Omalizumab was first used by Nadeau et al. as adjuvant therapy to OIT in 2011 in a pilot study of milk-allergic individuals (184). Since then, adjunctive omalizumab with OIT have been conducted for egg (190), peanut (191), sesame (192), and for multiple FA s (186). A prospective randomized controlled trial (RCT) of OIT combined with omalizumab using microwave heated cow’s milk was shown to help induce desensitization for children with high-risk cow’s milk allergy. The study was prematurely discontinued due to overwhelming superiority of the active group over the group avoiding cow’s milk (193). Another RCT of milk OIT plus omalizumab significant improved safety and time in achieving the maintenance dose but did not find increased success in desensitization or remission in patients (194). Results of a randomized, double-blind, placebo-controlled, multicenter trial (PRROTECT) of peanut allergy found that twelve weeks after stopping omalizumab, 76% and 12.5% of patients in the omalizumab and placebo arm, respectively, passed the 4000 mg food challenge. Although the overall reaction rates were not significantly different in the omalizumab versus the placebo arm; however, the omalizumab-treated subjects were exposed to much higher doses of peanut than the placebo group (195).A systematic review and meta-analysis of omalizumab in IgE-mediated FA found that omalizumab in conjunction with OIT significantly increased the tolerated dose of multiple foods, desensitization, and improved QoL. Immunological changes with increases in IgG4 are observed. There were no major safety concerns (185).

Dupilumab is also being evaluated as adjunctive therapy for FA in clinical trials for milk OIT (NCT04148352), peanut OIT (NCT03682770), and with omalizumab in multi- (NCT03679676).

### OIT and FA herbal formula-2 (FAHF-2)

In a murine model, FA Herbal Formula-2 (FAHF-2), which is a 9-herb formula based on traditional Chinese medicine, has been shown to blocks peanut-induced anaphylaxis (196). Additionally, in a phase I study FAHF-2was found to be safe and well tolerated, with favorable *in vitro* immunomodulatory effects (197). A double-blind, placebo-controlled study of enhanced, butanol purified FAHF-2 (E-B-FAHF-2) in combination with omalizumab-facilitated multiallergen OIT found that 63.6% of patients were desensitized to 4444 mg of protein for each allergen at 26 months and remission was achieved in about a quarter of the patients at 29 months, with no difference between the treatment groups. There were no differences in adverse events between the treatment groups or with adherence (>85%) to study medications. Overall, omalizumab-facilitated multifood OIT was safe and effective, and remission was achieved in about a quarter of subjects. However, outcomes were not improved by the addition of E-B-FAHF-2 (198).

### OIT with probiotics

Previous studies have suggested that probiotics may be effective in inducing remission. In a placebo-controlled study, children were treated with peanut OIT and the probiotic *Lactobacillus rhamnosus* for 18 months. Two to five weeks after treatment cessation, remission was achieved in 82.1% of children who received OIT plus probiotics compared with just 3.6% in the placebo group (199). Seventy percent of those who achieved remission in the active group were still tolerant to peanut at 4-year follow-up (200). As a follow-up, a multicenter, randomized, phase 2b trial randomized children to one of three groups: probiotic and peanut oral immunotherapy (PPOIT), placebo probiotic and peanut oral immunotherapy (OIT), or placebo probiotic and placebo OIT (placebo) for 18 months and then were followed up until 12 months after treatment completion. However, the study found no observable differences between the PPOIT and OIT groups in achieving remission (46% and 51%, respectively). Remission in the placebo group was 2% (201).

### Other therapies

Research on a number of novel treatments for FA are underway. These include IgE disruptors, vaccines, and Janus kinase (JAK) and Bruton’s tyrosine kinase (BTK) inhibitors.

### Vaccines

A DNA vaccine and a peptide vaccine are currently under investigation for FA. The vaccine is injected intradermally and is designed to train the immune system to desensitize individuals to the relevant allergy. In a murine shrimp allergy model, a Lit-LAMP-DNA-vaccine encoding multivalent shrimp antigens (Lit v (Litopenaeus vannamei; Whiteleg shrimp) 1, Lit v4, and Lit v3) and a lysosomal-associated membrane protein (LAMP) suppressed anaphylactic reactions and mast cell activation with the production of antigen-specific IgG2a. When plasma was transferred from mice previously vaccinated with the Lit-LAMP-DNA-vaccine, the suppression effect was also observed (202).

Clinical trials using LAMP vaccines have been conducted for those with peanut allergy or those with Japanese red cedar allergy (203, 204). A study evaluating the safety, tolerability, and immunogenicity of a peanut DNA vaccine (ASP0892) in adult with peanut allergy found that ASP0892 was well tolerated. However, although allergen-specific IgG and/or IgG4 increased, the increases were modest and not clinically relevant (205).

PVX108 is a peptide vaccine formulated for treatment of peanut allergy. It comprises of seven short peptides, which representing immunodominant T-cell epitopes of major peanut allergens. Preliminary results suggest that PVX108 is safe and tolerable in patients with peanut allergy (206). In a randomized, double-blind, placebo-controlled trial, PVX108 was safe and well tolerated in peanut allergic individuals with negligible activation of peanut-sensitized basophils (207).

### IgE: FcϵRI complex disruptors

DARPins (designed ankyrin repeat proteins) are emerging as potential therapeutics for allergy as they can be designed to disrupt the IgE: FcϵRI complex on mast cells and basophils. They are a novel class of small, single domain proteins which can be selected to bind any given target protein with high affinity and specificity. They provide an alternative to antibodies for targeted therapy. Their advantages over antibodies include their small size, stability, and low aggregation tendency, and ease of production (208). As demonstrated in *in vitro* and mouse models, DARPins reduce both free and mast and basophil-bound IgE thus decrease the risk of anaphylaxis via degranulation by mast cells and basophils (209).

### Nanoparticle

Nanoparticles by encapsulating allergens protect them from acidic and enzymatic degradation and enable targeted therapy. In a murine model, masking of allergens in poly(lactide-co-glycolide) (PLG) nanoscale particles safely attenuated anaphylactic response in murine models of peanut allergy. Application of 2-3 doses, without the need for dose escalation, suppressed mast cell degranulation (210). In another murine model of cow’s milk allergy, oral pretreatment with β-lactoglobulin derived peptide and CpG co-encapsulated in poly (lactic-co-glycolic acid) nanoparticles prior to sensitizations attenuated cow’s milk allergy development (211).

### Janus kinase and Bruton’s tyrosine kinase inhibitors

Janus kinases (JAK) are a family of intracellular, non-receptor tyrosine kinases that transduce cytokine-mediated signals via the JAK-STAT pathway (212). A number of JAK/STAT inhibitors (JAK inhibitors) have been developed and a few have been approved for treatment of atopic dermatitis. A study using whole blood from peanut allergic individuals, found that inhibition of JAK1 (which suppresses IL-4, IL-13, IL-9, and TSLP signaling) by abrocitinib led to the inhibition of allergen-specific basophil activation, dose-dependent inhibition of TH_2_ cytokine levels, and spared Treg cell activation (213). The drug is currently in clinical trials for the treatment of FA (NCT05069831).

Bruton’s tyrosine kinase (BTK) inhibitors block signaling downstream of the FceRI (214). In a small phase 2 trial of acalabrutinib in patients with peanut allergy found clinically relevant increases in tolerance to peanuts. Side effects were transient and not serious (215). Ibrutinib, another BTK inhibitor, has also evaluated in a small number of patients with peanut allergy. Short-term treatment of the drug suppressed skin test responses and significantly reduced basophil activation (216).

## Conclusion and future directions

In the last few decades, we have made great strides in understanding the pathogenesis of IgE mediated FA, but many gaps in research and treatment remain. There is a need for accurate and sensitive biomarkers for diagnosis of FA, enable identification of those at risk of severe reaction, and monitor efficiency of treatments. Although In recent years, a number of studies using high throughput omic technologies to understand molecular and cellular differences between those with and without FA or food sensitization are ongoing and are overcoming these limitations (217, 218). Artificial Intelligence (AI) technologies may also eliminate the need for invasive and potentially dangerous tests. In the future, it is likely that BATs, epitope mapping, and omics methods, along with sIgE tests and medical history, will be used to generate rich, high-dimensional data sets which can be synthesized by AI to build patient-specific models, guiding doctors and patients in selecting optimal FA management strategies. AI-enabled technologies have the potential to make routine use of OFCs and SPTs obsolete (219). Microfluidic methods are also being explored for precision diagnostics, including multiplexing the detection of multiple biomarkers, sampling of tissue-resident cytokines and immune cells, and multi-organ-on-a-chip technology (220).The ultimate goal is to understand the causes of immune dysfunction in order to prevent skewing of immune cells towards hypersensitivity.

The approval of two drugs for FA, omalizumab and Palforzia, are major milestones in FA treatment. However, neither of them offer a cure. Omalizumab desensitizes an individual to FA, but treatment is to be used in conjunction with food allergen avoidance with benefits limited to reduction in risk of severe reaction on accidental ingestion. While omalizumab can be used in patients with any FA, Palforzia is limited to those with peanut allergy. An additional drawback is that desensitization with these drugs is not permanent. Research needs to work towards a drug that can simultaneously desensitize patients to multiple allergens and offer a cure, a permanent state of immune tolerance, rather than a temporary period of desensitization. As detailed above, there is a broad range of treatment types that are being evaluated in addition to immunotherapy, including vaccines, nanoparticles, biologics, Janus Kinase and Bruton’s tyrosine kinase inhibitors, or DARPins, many which show promise.
